# Case Report: Diagnosis of Human Alveolar Echinococcosis via Next-Generation Sequencing Analysis

**DOI:** 10.3389/fgene.2021.666225

**Published:** 2021-07-09

**Authors:** Ke Li, Yubao Ma, Rui Ban, Qiang Shi

**Affiliations:** ^1^Department of Neurology, The Second Medical Center, Chinese PLA General Hospital, Beijing, China; ^2^Department of Neurology, The First Medical Center, Chinese PLA General Hospital, Beijing, China; ^3^Department of Neurology, Beijing Children's Hospital, Capital Medical University, National Center for Children's Health, Beijing, China

**Keywords:** alveolar echinococcosis, next-generation sequencing, adrenal gland, *Echinococcus multilocularis*, diagnose

## Abstract

**Introduction:** Alveolar echinococcosis (AE) is a rare parasitic disease caused by the infection of *Echinococcus multilocularis*. AE may mimic malignancy both in clinical presentation and radiological imaging, which is often misdiagnosed as metastatic tumor. Recently, next-generation sequencing (NGS) technologies are increasingly being used to address a diverse range of biological questions. Here, we describe a rare case of alveolar echinococcosis diagnosed by pan-pathogen screening, using next-generation sequencing. To the best of our knowledge, this is the first reported case of AE which was definitely diagnosed relying NGS of cerebrospinal fluid (CSF).

**Case Presentation:** A 33-year-old man presented with repeat seizure and progressive headache for six months. Head magnetic resonance imaging (MRI) showed multiple masses with edema. Lung and abdominal computer tomography (CT) revealed multiple masses in bilateral lung, liver and the right adrenal gland. Bacterial, tuberculosis and fungal infection were excluded by CSF examination. Repeated target biopsy on the masses in the lung and liver showed as fibrous connective tissue without positive findings. NGS of CSF was performed and detected nucleic acid sequences of *E. multilocularis*. Consequently, the patient has accepted 1-year albendazole therapy. His case was followed up through imaging procedures.

**Conclusion:** The next-generation sequencing of CSF is a reliable and sensitive diagnostic method for the detection of pathogenic microorganisms, and may allow the accurate diagnosis of alveolar echinococcosis. In view of this case, we recommend NGS as a potential tool for diagnosis of cerebral AE, especially if repeated biopsies are negative.

## Introduction

Brain alveolar echinococcosis is a fatal parasitic disease caused by *Echinococcus multilocularis* (Deplazes et al., 2017), which is often misdiagnosed as metastatic tumor or intracranial tuberculosis. Diagnosis of AE relies on clinical presentation, imaging examinations, serological test and biopsy if available. However, metastatic tumors and AE are difficult to differentiate through imaging examinations. In addition, serological test sometimes results in biologically false-positive results. Herein, we present a rare case of cerebral AE which was finally confirmed by NGS. This is the first reported case which is conclusively diagnosed as cerebral AE by NGS of CSF.

## Case Presentation

**Figure 1:**
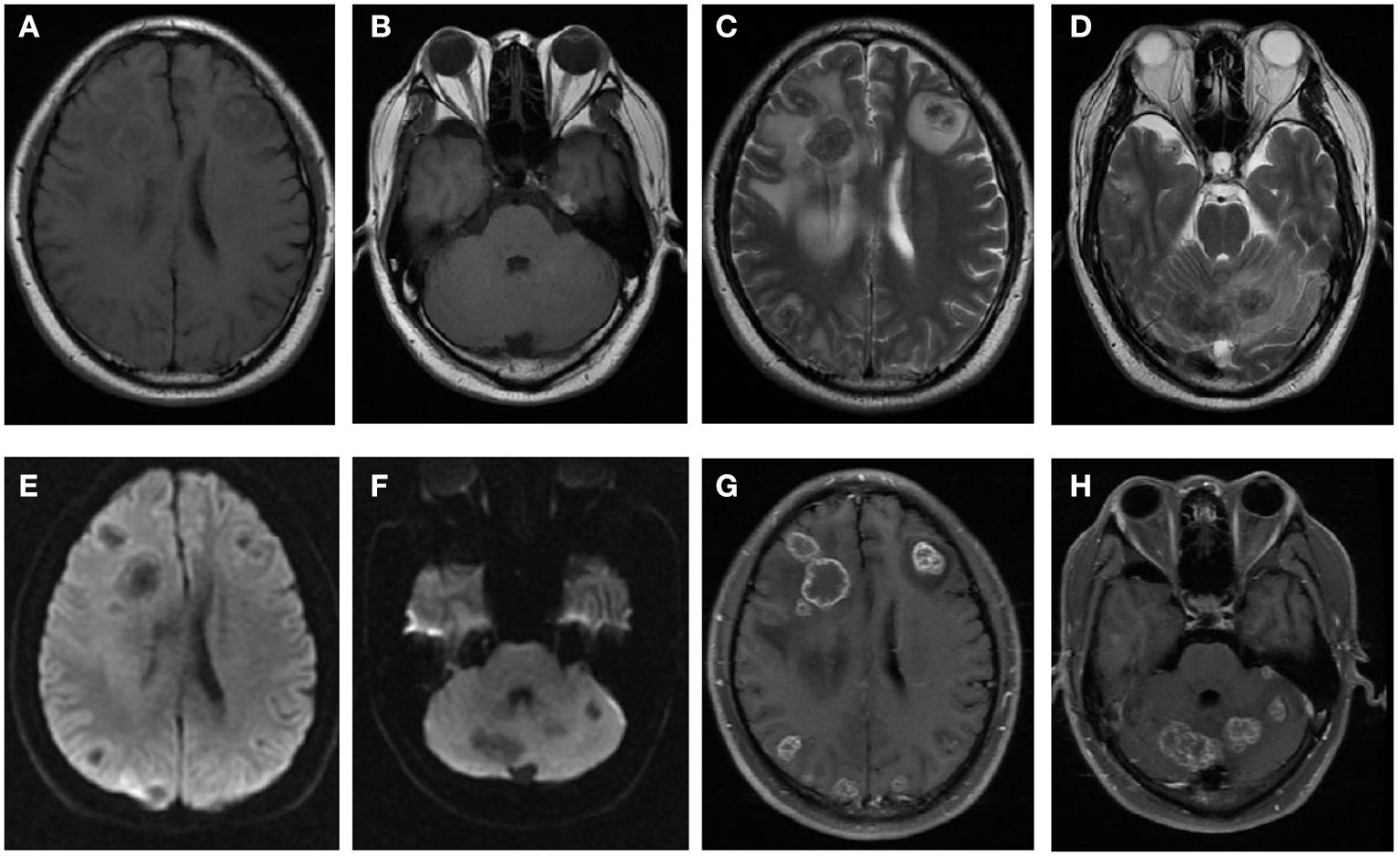
Brain MRI images of the patient. Multiple lesions revealed with isointensity on T1WI **(A,B)**, hypointensity on T2WI **(C,D)** surrounded by edema, hypointensity on DWI **(E,F)** and irregular ring enhancement after injection of Gd-DTPA **(G,H)**.

**Figure 2:**
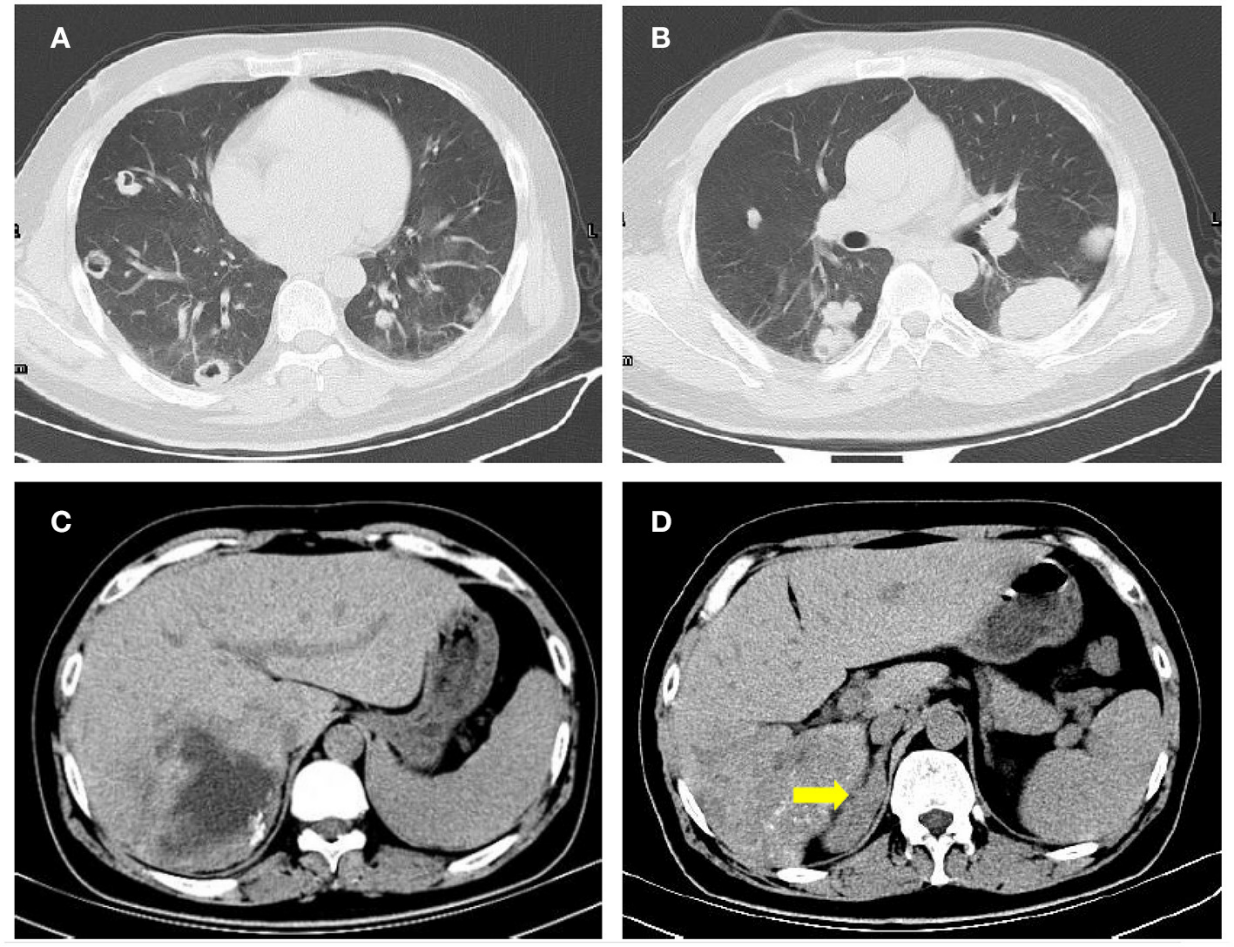
Lung and abdominal CT images of the patient. Multiple Lesions were found in bilateral lung **(A,B)** and the liver **(C)**. The right adrenal gland (yellow arrow) was obviously thickened **(D)**.

**Figure 3:**
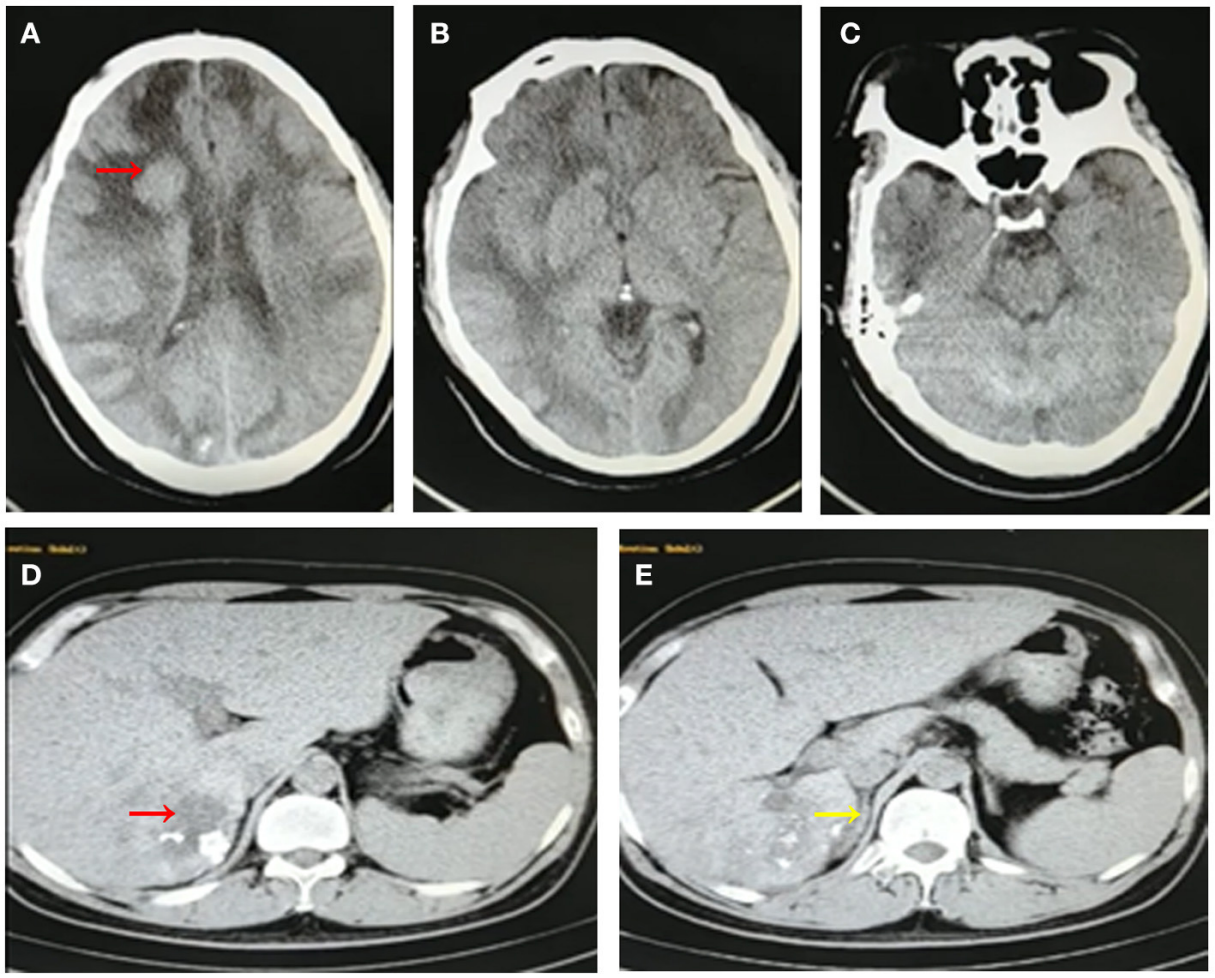
Follow-up CT images of the patient after 1-year albendazole therapy. Brain CT images revealed multiple lesions has slightly shrink and surrounding edema was slightly relieved **(A–C)**. Abdominal CT images showed that the lesion in liver was obviously diminished accompanied with calcification **(D,E)**.

A 33-year-old man presented repeat seizures and progressive headache for six months. A complete blood count (CBC) showed: hemoglobin 123g/L, white blood cell count 7.73 × 10^9^/L, and percentage of eosinophil 0.009. Liver function showed: alanine transaminase (ALT) 92.8 U/L, aspartate aminotransferase (AST) 44.9U/L, alkaline phosphatase (ALP) 135.1U/L, γ-glutamyl transferase (γ-GT) 313.4 U/L, total bilirubin (TBil) 22.7 mmol/L, direct bilirubin (DBil) 19.4 mmol/L. Head MRI (Figure 1) showed multiple masses with edema. Lung and abdominal CT (Figure 2) presented with several lesions in bilateral lungs, liver and right adrenal gland. Bacterial, tuberculosis and fungal infection were excluded by CSF examination. Serological evaluation of multiple parasite antigen by ELISA were applied. Cysticercosis Immunoglobulin G (IgG) and liver hydatid IgG antibody were both positive. Repeated target biopsy on the masses in the lung and liver showed fibrous connective tissue without positive findings. In addition, we performed positron emission tomography with fluorodeoxyglucose integrated with CT (^18^F-FDG PET/CT) that showed uptake in all masses with a maximum standardized uptake value of 7.2. Accordingly, it was still hard to draw a definitive pathogenic diagnosis. Therefore, the next-generation sequencing of CSF was performed. The CSF was collected according to standard procedures, and DNA was extracted directly from the sample with TIANamp Micro DNA Kit. The extracted DNA was sonicated to a size of 200–300 bp (Bioruptor Pico protocols). The DNA libraries were constructed and sequencing using the BGISEQ-100 platform. After removing human sequences, the remaining sequencing data were aligned to the microbial databases and detected 161 nucleic acid sequences of *E. multilocularis* (Table 1, *E. multilocularis* sequences which detected were provided as Supplementary Material). On this basis, the patient was diagnosed as having AE. Consequently, the patient was recommended a 1-year albendazole therapy. During 1 year of follow-up, symptoms and neurological signs were not aggravated, with decreased seizure frequency. Follow-up CT after 1 year of albendazole treatment revealed slightly decreased multiple lesions and partly relieved surrounding edema in brain. The lesion in the liver (red arrow) was evidently diminished and calcification was slightly increased. The thickened right adrenal gland (yellow arrow) has obviously decreased in size (Figure 3).

**Table 1:** List of parasites detected.

**Genus**		**Species**	
**Name**	**Numbers of sequences detected**	**Name**	**Numbers of sequences detected**
*Echinococcus*	318	*Echinococcus multilocularis*	161

## Discussion and Conclusions

We reported a special human alveolar echinococcosis case involving the brain, lung, liver, adrenal gland, which was confirmed by NGS. AE is endemic in certain parts of the world, especially in Europe, Northern America, and Central Asia (Deplazes et al., 2017). A recent meta-analysis indicated that the pooled prevalence of AE in China was 0.96% (Wang et al., 2020). In China, the AE endemic area is restricted to the northwest region (Qian et al., 2017), for example, Qinghai province and Gansu province. In most cases, AE is initially located in the liver and spreads into other organs by infiltration or metastasis formation. Extrahepatic lesions are usually located in the lung and brain (Tappe et al., 2008). Adrenal AE, however, is rare, with only 9 cases reported in the literature so far (Huang and Zheng, 2013; Spahn et al., 2016; Seidel et al., 2017). Our case suggests that AE may involve all parts of the body. We should suspect the diagnosis of AE in a patient with exposure history of parasite and a mass in the adrenal gland.

The diagnosis of AE is complicated by diverse clinical features and mimicking of differential diagnoses. For this case, the patient's biopsy results were negative. Therefore, a biopsy is likely to have lower sensitivity for the diagnosis of AE due to limited tissue specimens. Imaging findings of multiple lesions are helpful in differential diagnosis; however, it can be misinterpreted as other metastatic tumors even by experienced radiologists due to their limited awareness of this condition. Another difficulty in the differential diagnosis of AE and cysticercosis is that serologic test revealed that both of cysticercosis IgG and liver hydatid IgG were all positive. Is it a co-infection or immunological cross-reaction? We prefer immunological cross-reaction since there are common antigens between *Cysticercus cellulosae* and *E. multilocularis*. While mixed infection of 2 pathogens is extremely rare. Furthermore, NGS detected nucleic acid sequences of *E. multilocularis* without sequences of *C. cellulose*, which conclusively exclude cysticercosis.

Finally, AE diagnosis has been confirmed by NGS for this patient. NGS technologies are increasingly being used to address a diverse range of biological and epidemiological questions since NGS provides pathogen identification without prior target knowledge. In addition, other genetic diagnostic methods, such as PCR, is not applicable for screening of rare and unknown pathogens. We believe that this is the first reported case of AE which was definitely diagnosed relying next-generation sequencing of CSF. Clinicians should promptly recognize that NGS of CSF might be provided as a potential test for detecting cerebral AE.

The limitation of this study includes small sample size. Therefore, if there are similar cases in the future, we should expand the sample size and validate the accuracy of NGS method. In conclusion, the NGS of CSF is a reliable and sensitive diagnostic method to detect pathogenic microorganisms, which may allow accurate diagnoses of cerebral AE.

## Data Availability Statement

The original contributions presented in the study are included in the article/Supplementary Material, further inquiries can be directed to the corresponding author/s.

## Ethics Statement

The studies involving human participants were reviewed and approved by This work was supported by National Natural Science Foundation of China (No. 81601086, 81771358). The patients/participants provided their written informed consent to participate in this study. Written informed consent was obtained from the individual(s) for the publication of any potentially identifiable images or data included in this article.

## Author Contributions

All co-authors have seen and agree with the contents of the manuscript, that the ICMJE requirements for authorship have been met, and that each author believes that the manuscript represents honest work. All authors contributed to the article and approved the submitted version.

## Conflict of Interest

The authors declare that the research was conducted in the absence of any commercial or financial relationships that could be construed as a potential conflict of interest.
